# Chromosome 8q as the most frequent target for amplification in early gastric carcinoma

**DOI:** 10.3892/ol.2014.1849

**Published:** 2014-02-03

**Authors:** JI UN KANG

**Affiliations:** Department of Biomedical Laboratory Science, Korea Nazarene University, Cheonan 330-718, Republic of Korea

**Keywords:** gastric carcinoma, microarray CGH, copy number gains, high-level amplifications

## Abstract

Early gastric carcinoma (GC) is considered to be a curable cancer, as it progresses to the advanced stage following varying durations. Understanding the early stage of GC may provide an insight into its pathogenesis and contribute to reducing the mortality rate of this disease. To investigate the genomic aberrations associated with 22 cases of early GC, high-density microarray comparative genomic hybridization was performed in the present study. The most notable finding was copy number gains (log_2_ ratio >0.25) on the long arm of chromosome 8, which occurred in 77.3% (17/22) of GC cases, and the delineated minimal common region was 8q22.1-q24.3. More specifically, two amplified (log_2_ ratio >1) loci in the 8q22.1-q24.3 region were detected in 18.2% (4/22) of GC cases. The first loci covered a region of 102.4–107.9 kb, mapping on 8q22.3-q23.1, and comprised the transcription factor CP2-like 3 gene. The second loci, spanning 128.7–145.7 kb on 8q24.21-q24.3, comprised the representative oncogene of myelocytomatosis. Furthermore, the following possible target genes that were not previously considered to play a pathogenic role in GC were identified: Plasmacytoma variant translocation 1, cysteine/histidine rich 1, kinesin family member C2, forkhead box H1, protein phosphatase 1 regulatory subunit 16A, glutamic-pyruvate transaminase, *LOC113655* and RecQ protein-like 4. In the present study, previous findings showing that 8q mutations accumulate early during the multistage pathogenesis of GC were confirmed and expanded upon. The confirmation of previously reported 8q gains and the identification of novel target genes at 8q22.1-q24.3 amplified chromosomal sites should aid in improving our understanding of the molecular mechanisms underlying the tumorigenesis of early GC.

## Introduction

Eradication of early gastric carcinoma (GC) could contribute to a reduction in the mortality of GC, given that most early GCs progress to become advanced GCs (1). Therapeutic interventions for late-stage GC are usually limited to non-curative gastrectomy, lymphadenectomy and post-operative chemoradiotherapy (2). The five-year relative survival rates for GC patients are <30% in the majority of countries (3). Hence, the identification of novel biomarkers is of great clinical importance for early diagnosis, targeted treatment and prognosis evaluation in GCs.

Gastric tumorigenesis is a heterogeneous process that occurs following a series of clonal molecular genetic alterations, including genomic gains and losses, particularly deletion of tumor-suppressor genes and amplification of oncogenes. Unveiling abnormalities of specific genes may offer novel insights into the mechanisms of local growth or the metastatic potential of early GCs, and allow patients to be stratified into different risk categories or be treated with novel options for targeted therapy (4).

Cytogenetic studies have been performed to evaluate genetic alterations associated with early GCs (1,2,4,5). Defining the genetic instability aids in the identification of the tumor-specific signatures involved in the initiation and progression of GCs, and thus aids in locating genomic biomarkers for the early detection of GC (5). However, the molecular mechanism of GC development remains to be understood, and identification of the predictive markers in the early stage is crucial. There is a critical requirement for identifying biomarkers for early detection and novel treatments for GC. Therefore, in the present study, whole genome array comparative genomic hybridization (CGH) was conducted to investigate DNA copy number alterations and new candidate genes that may be indicative and specific for early GC.

## Materials and methods

### Study materials

A total of 22 gastric tumor samples were obtained from patients treated at the Department of General Surgery of Chungnam National University Hospital in Taejeon, South Korea. None of these patients had received pre-operative chemotherapy or radiation. The stage of disease was based on the tumor-node-metastasis classification using the Union International Cancer Center staging system. The original diagnostic material of all patients was reviewed to verify the original histopathological diagnosis and staging according to the World Health Organization classification system (6). The present study was reviewed and approved by the Institutional Review Board of the Chungnam National University Hospital, and written informed consent was obtained from each patient according to the institutional regulations.

### Array-CGH experiment

DNA isolation was performed using a DNA isolation kit according to the manufacturer’s instructions (Promega, Madison, WI, USA), with certain modifications as previously described (7,8). Array-CGH was conducted using the MacArray™ Karyo 1.4 K BAC-chip (Macrogen, Seoul, Korea) (9–11) according to the manufacturer’s instructions and as described in our previous studies (12,13). Briefly, all clones were two-end sequenced using an ABI PRISM 3700 DNA Analyzer (Applied Biosystems, Foster City, CA, USA), and their sequences were blasted (BLAST; http://blast.ncbi.nlm.nih.gov/Blast.cgi).

Hybridizations were carried out using a standard direct method as previously described (14,15). Briefly, 500 ng normal male DNA (reference) and digested tumor DNA (test) was labeled with Cy5-dCTP and Cy3-dCTP, respectively, by random primed labeling (Array CGH Genomic Labeling System; Invitrogen, Carlsbad, CA, USA). Hybridizations were performed in a sealed chamber for 48 h at 37°C. Subsequent to hybridization, slides were washed according to the manufacturer’s instructions and immediately scanned on a GenePix 4200A two-color fluorescence scanner (Axon Instruments, Union City, CA, USA). The acquired images were analyzed using GenePix Pro 4.1 imaging software (Axon Instruments).

### Array CGH data analysis

Breakpoint detection and status assignment of the genomic regions were performed using a Gaussian model-based approach (GLAD). The median of the signal ratio (test signal/reference signal) of each triplicated spot was defined as a gain or a loss when it was >0.25 or <−0.25, respectively. High-level amplification of clones was defined when their intensity ratios were >1.0 in log_2_ scale, and vice versa for homozygous deletion. The threshold value was determined empirically as a value 3-fold that of the standard deviations calculated from 30 normal males and females in hybridization experiments. The R 2.2.1 package of the Bioconductor Project (http://www.bioconductor.org) was used for the detection of the frequency of gain or loss and for statistical analysis. The Benjamini-Hochberg false discovery rate was applied for multiple testing corrections for the high number of false-positive calls.

## Results

### Genome wide array analysis in early GC cases

In total, 22 tumor samples were assessed by high-resolution array CGH to investigate DNA copy number alterations and new candidate genes associated with early GCs. The majority of clones were frequently gained (log_2_ ratio >0.25) or lost (log_2_ ratio <−0.25), with 97.5% of the clones being gained or lost in 62.7% of the cases. As the first step of the analysis, chromosome 8q, the most frequently gained [77.3% (17/22)] and amplified [log_2_ ratio >1, 18.2% (4/22)] region in GC cases, was focused upon. There were high-level amplifications on chromosome 8 at four distinct loci, centered at 106.4, 89.6, 77.6 and 110.5 Mb (containing *MYC*). The most common region with copy number increase in GC cases was more clearly defined and narrowed down to 8q22.1-q24.3, encompassing BAC177_N09 to BAC145_J12. A list of the delineations of the 8q22.1-q24.3 chromosomal region and possible target genes of GC is presented in Table I.

### Chromosomal alterations of 8q22.1-q24.3 are the most common genetic changes in early GCs

In the 8q22.1-q24.3 region, two common regions of alterations across the chromosome were further clarified. The first loci spanned 96.3–116.8 kb, and was mapped at the 8q22.1-q23.3 region. Notably, two amplified loci in these regions were identified in 9.1% (2/22) of the cases. One locus at 8q22.3 contained amplified clones covering a region of ~106.4 kb, and comprised the transcription factor CP2-like 3 (*TFCP2L3*) gene and the other locus spanning ~89.6 kb on 8q23.1, without associated genes. The median span of the copy number amplifications was 16.8 kb (range, 89.6–106.4 kb), and all amplifications were located between BAC157_F12 and BAC169_B05.

**Figure 1:**
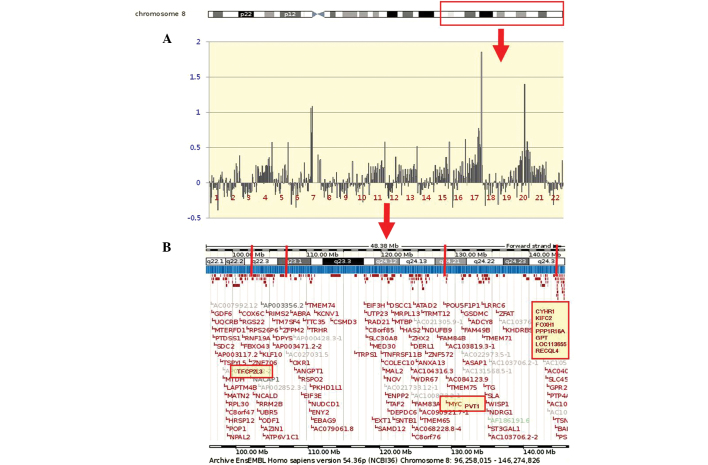
(A) An example of an individual profile at the 8q22.1-q24.3 region in the 22 GC cases. High-level amplifications are clearly seen in cases 7, 17 and 20. Cytobands in the ideogram are shown in the upper image. (B) The schematic presentation of cytogenetic bands, and a map position from the UCSC genome browser is shown below the plot. The candidate target genes (*TFCP2L3, MYC, PVT1, CYHR1, KIFC2, FOXH1, PPP1R16A, GPT, LOC113655* and *RECQL4*) at the 8q22.1-q24.3 region are shaded in yellow. GC, gastric carcinoma; UCSC, University of California Santa Cruz; *TFCP2L3*, transcription factor CP2-like 3; *MYC*, myelocytomatosis; *PVT1*, plasmacytoma variant translocation 1; *CYHR1*, cysteine/histidine rich 1; *KIFC2*, kinesin family member C2; *FOXH1*, forkhead box H1; *PPP1R16A*, protein phosphatase 1 regulatory subunit 16A; *GPT*, glutamic-pyruvate transaminase; *RECQl4*, RecQ protein-like 4.

**Figure 2:**
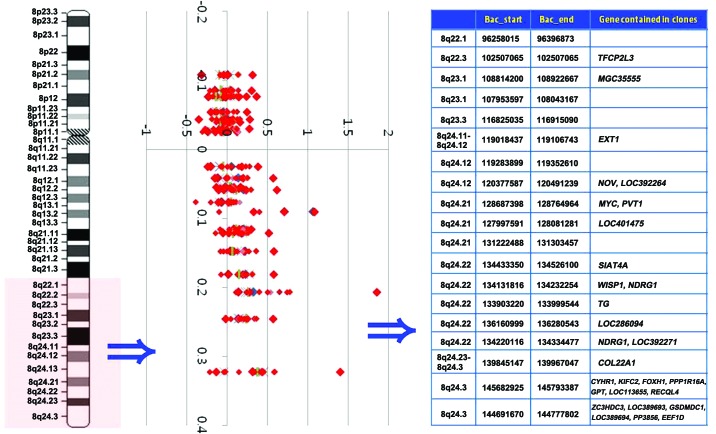
Weighted frequency (%) diagram of the 8q22.1-q24.3 region in GC cases. In the intensity ratio profiles, the Y-axis represents the map position of the corresponding clone and the intensity ratios are assigned to the X-axis. Cytobands in the ideogram are shown on the left. Genes contained in clones are shown on the right. GC, gastric carcinoma; *MYC*, myelocytomatosis; *PVT1*, plasmacytoma variant translocation 1; *CYHR1*, cysteine/histidine rich 1; *KIFC2*, kinesin family member C2; *FOXH1*, forkhead box H1; *PPP1R16A*, protein phosphatase 1 regulatory subunit 16A; *GPT*, glutamic-pyruvate transaminase; *RECQl4*, RecQ protein-like 4.

The second loci spanned 119.0–144.7 kb on 8q24.11-q24.3, and this region contained 27 possible target genes according to the information archived from human genome databases (http://genome.ucsc.edu/). Significantly, a high frequency of copy number gains (log_2_ ratio >0.25) and high-level gains (log_2_ ratio >0.5) in the 8q24.11-q24.3 region were detected in 77.3 (17/22) and 36.4% (8/22) of the GC cases, respectively. More specifically, two amplified (log_2_ ratio >1) loci in these regions were noted in 9.1% of the GC cases. One locus at 8q24.21 comprised the representative oncogenes of myelocytomatosis (*MYC*) and plasmacytoma variant translocation 1 (*PVT1*) (4.5%). The other locus spanning ~110.5 kb on 8q24.3 was found to contain cysteine/histidine rich 1 (*CYHR1*), kinesin family member C2 (*KIFC2*), forkhead box H1 (*FOXH1*), protein phosphatase 1 regulatory subunit 16A (*PPP1R16A*), glutamic-pyruvate transaminase (*GPT*), *LOC113655* and RecQ protein-like 4 (*RECQL4*) genes with the highest level of amplification. The median span of the copy number amplifications of the 8q24.11-q24.3 region was 32.9 kb (range, 77.6–110.5 kb), and all amplifications were located between BAC192_N08 and BAC145_J11. An example of an individual profile at the 8q22.1-q24.3 region in the 22 GC cases is shown in Fig. 1. A representative weighted frequency (%) diagram with high-level amplifications in the 8q22.1-q24.3 region for all 22 GC cases is displayed in Fig. 2.

## Discussion

In the present study, the most notable finding was the frequent genetic abnormality of chromosome 8q in early GC cases. The long arm of chromosome 8 has long been suspected to contain critical oncogenes that lead to numerous cancer types, including GC (14–17). Abi-Ayad *et al* (16) reported that copy number gains of the 8q arm (70%) or the entirety of chromosome 8 were the most frequent imbalance in melanoma. de Krijger *et al* (17) also reported that chromosome 8q gains were the most frequent genetic abnormality in pleuropulmonary blastoma. Notably, the two surviving patients in the series exhibited gains of chromosome 8q material as the only genetic abnormality. Furthermore, Buffart *et al* (14) documented copy number gains of genes on chromosome 8q in 9.5–73.0% of the GC cases, with the highest frequency of gains in *MYC* (73.0%). More recently, Cheng *et al* (2) documented the highest frequency (70%) of copy number gains at 8q in GCs by array CGH analysis. By combining the results of the present study with those in other studies, it is indicated that copy number gains on chromosome 8q occur as an early event in the multistage development of various tumors, including GC, which commences in the mildly abnormal epithelium.

More specifically, the smallest region of overlap, the 8q22.11-q24.3 region, was determined. A high frequency of single copy number gains (log_2_ ratio >0.25) and high-level gains (log_2_ ratio >0.5) in this region was detected in 77.3 and 36.4% of the cases, respectively. Notably, two amplified (log_2_ ratio >1) loci in these regions were detected in 18.2% (4/22) of the GC cases. One locus on 8q24.3 was found to contain *CYHR1, KIFC2, FOXH1, PPP1R16A, GPT, LOC113655* and *RECQL4* genes, placing the highest level of amplifications in the GC cases. To the best of our knowledge, the involvement of these genes in the pathogenesis of GC has not been described previously; however, genetic mutations of these genes are commonly found in various types of cancers. Katoh *et al* (15) reported high-level amplification and overexpression of the *FOXA1* gene in esophageal and lung cancer. Upregulation of the *FOXA1* gene in pancreatic cancer and basal cell carcinoma, due to the transcriptional regulation by the Sonic Hedgehog (SHH) pathway, has also been documented. Furthermore, overexpression of the *RECQL4* gene with chromosomal aberrations and instability in osteosarcoma has also been reported (18,19). These findings indicate that chromosomal alterations of these developmental genes may contribute in playing a role in the underlying mechanism of cancer development.

The other amplified locus at 8q24.21 comprised the representative oncogene of *MYC*. Several experimental studies have shown that *MYC* amplification or overexpression is observed in early GC patients when tumor invasion is confined to the mucosa or submucosa, regardless of the presence of lymph node metastasis (2,18–22). In the study by Onoda *et al* (20), *MYC* expression was found to be more frequent and stronger in early lesions compared with advanced lesions, and Ishii *et al* (21) also documented increased *MYC* expression in early GCs in comparison with decreased *MYC* expression in late-stage GCs. Based on these findings and the data from the present study, *MYC* genetic aberrations may be an early event in the pathogenesis of gastric carcinogenesis, and the detection of *MYC* locus amplification could be used as an auxiliary tool in GC diagnosis and as a predictor of GC aggressiveness (22).

In addition, one potential oncogene candidate of *PVT1* was identified as a potential target within the 8q24.21 amplicon. *PVT1* encodes a non-coding RNA and is a host gene for several miRNAs, namely hsa-miR-1204, 1205, 1206 and 1207 (23). Although involvement of the *PVT1* gene in the pathogenesis of GC has not been mentioned thus far, genetic mutations of the *PVT1* gene have been consistently reported in multiple types of tumors (23–26). Nagoshi *et al* (24) indicated that *PVT1* rearrangements represent a novel molecular paradigm underlying the pathology of 8q24.21 rearrangement-positive multiple myeloma. *PVT1* also showed increased expression in prostate cell lines compared to normal prostate tissue (25). In addition, Meyer *et al* (23) reported that the risk locus can interact with two downstream genes, *MYC* and *PVT1*, and that *PVT1*, a novel target gene candidate, regulates the 8q24 risk region. Additionally, an association between genetic variants within *PVT1* and Hodgkin’s lymphoma has also been postulated (26). Taken together, the results of the present study and the findings of other studies also present evidence that *PVT1* is a new target gene candidate, regulated by the 8q24 risk region, and that it could be defined as an independent target region for chromosome 8q amplifications in various tumors, including GCs. Further functional and biological studies are required to validate and evaluate the role of the *PVT1* gene as a novel candidate oncogene in GC in larger series and on multiple samples.

In summary, the present study confirms and expands upon previous observations that 8q genetic mutations accumulate early during the multistage pathogenesis of GC. The confirmation of previously reported 8q gains and the identification of novel target genes at 8q22.1-q24.3 amplified chromosomal sites should provide important clues with regard to the genetic mechanisms of initiation and progression, and provide new insights into the clinical behavior and management of GC. Additional genome-wide studies with a larger number of patients are warranted to confirm the results of the present study and to improve our understanding of GC.

## Figures and Tables

**Table I:** Various chromosomal recurrent minimal regions of genetic alterations on the long arm of chromosome 8 in 22 GCs.

Regions	Gene contained in clones	% of gains^a^	% of amplifications^b^
8q21.1-q21.3	IL7, PMP2, FABP9, FABP4, MMP16, NBS1, DECR1	22.7 (5/22)	
8q22.1-q23.3	TFCP2L3, MGC35555	18.2 (4/22)	9.1 (2/22)
8q24.11-q24.3	EXT1, NOV, LOC392264, MYC, PVT1, LOC401475, SIAT4A, WISP1, NDRG1, TG, LOC286094, NDRG1, LOC392271, COL22A1, CYHR1, KIFC2, FOXH1, PPP1R16A, GPT, LOC113655, RECQL4, ZC3HDC3, LOC389693, GSDMDC1, LOC389694, PP3856, EEF1D	77.3 (17/22)	9.1 (2/22)

a Alterations were defined by log_2_ ratio thresholds of >0.25 for copy number gain.

b Alterations were defined by log_2_ ratio thresholds of 1 log_2_ ratio for high-level amplification.

Genomic positions were retrieved from the UCSC Genome browser web page [http://genome.cse.ucsc.edu; Build 36, March 2006 version (hg18)].

GC, gastric carcinoma; UCSC, University of California Santa Cruz.
